# Normal Variability of Weekly Musculoskeletal Screening Scores and the Influence of Training Load across an Australian Football League Season

**DOI:** 10.3389/fphys.2018.00144

**Published:** 2018-02-27

**Authors:** Alireza Esmaeili, Andrew M. Stewart, William G. Hopkins, George P. Elias, Brendan H. Lazarus, Amber E. Rowell, Robert J. Aughey

**Affiliations:** ^1^Institute of Sport, Exercise and Active Living, Victoria University, Melbourne, VIC, Australia; ^2^Western Bulldogs Football Club, Melbourne, VIC, Australia; ^3^Norwegian Defence Institute, Oslo, Norway; ^4^Collingwood Football Club, Melbourne, VIC, Australia; ^5^Melbourne Victory Football Club, Melbourne, VIC, Australia

**Keywords:** injury prevention, athlete monitoring, recovery, modeling, hamstring, groin, calf

## Abstract

**Aim:** The sit and reach test (S&R), dorsiflexion lunge test (DLT), and adductor squeeze test (AST) are commonly used in weekly musculoskeletal screening for athlete monitoring and injury prevention purposes. The aim of this study was to determine the normal week to week variability of the test scores, individual differences in variability, and the effects of training load on the scores.

**Methods:** Forty-four elite Australian rules footballers from one club completed the weekly screening tests on day 2 or 3 post-main training (pre-season) or post-match (in-season) over a 10 month season. Ratings of perceived exertion and session duration for all training sessions were used to derive various measures of training load via both simple summations and exponentially weighted moving averages. Data were analyzed via linear and quadratic mixed modeling and interpreted using magnitude-based inference.

**Results:** Substantial small to moderate variability was found for the tests at both season phases; for example over the in-season, the normal variability ±90% confidence limits were as follows: S&R ±1.01 cm, ±0.12; DLT ±0.48 cm, ±0.06; AST ±7.4%, ±0.6%. Small individual differences in variability existed for the S&R and AST (factor standard deviations between 1.31 and 1.66). All measures of training load had trivial effects on the screening scores.

**Conclusion:** A change in a test score larger than the normal variability is required to be considered a true change. Athlete monitoring and flagging systems need to account for the individual differences in variability. The tests are not sensitive to internal training load when conducted 2 or 3 days post-training or post-match, and the scores should be interpreted cautiously when used as measures of recovery.

## Introduction

Musculoskeletal screening refers to a series of tests designed to detect internal abnormalities that are associated with increased injury risk, or incomplete recovery from training or previous injuries (Dennis et al., 2008; Morgan et al., 2014; Bahr, 2016). The ultimate aim of screening is to implement effective interventions such as treatments, injury prevention exercises, or training modifications before an injury occurs (Bahr, 2016).

Pre-season (pre-participation) musculoskeletal screening is a widely studied approach where athletes are tested at the start of pre-season and then monitored prospectively for occurrence of injuries for the remainder of the season. Cut scores are then set with the aim of identifying athletes with high injury risk (Bahr, 2016). This approach has been criticized for its poor predictive ability and the risk of providing a false sense of security (Bahr, 2016; Whiteley, 2016). It has also been argued that pre-season test scores only represent the athlete's condition at that particular time which may vary throughout the season as a result of exposure to training and competition (Whiteley, 2016).

Repeated-measures or regular screening is another approach that involves frequently conducting testing and measuring the change in screening test scores (Paul et al., 2014). The rationale behind the repeated-measures format is that changes in screening scores better reflect the condition of athletes, how they are responding to training, and subsequent injury risk (Paul et al., 2014; Thorpe et al., 2017). The concept of repeated-measures testing and monitoring of athletes has been applied to the physiological, hormonal, biochemical, psychological, and neuromuscular measures of recovery (Taylor et al., 2012; Thorpe et al., 2017). The repeated-measures musculoskeletal screening strategy for athlete monitoring and injury prevention purposes is gaining momentum in professional sports, however, the underlying evidence to support this approach is very limited.

Injuries to hamstring, groin, and calf muscles are among the most common injuries in Australian football, and the musculoskeletal screening tests implemented by Australian Football League (AFL) clubs attempt to monitor some of the intrinsic risk factors associated with these injuries (Gabbe et al., 2004; Orchard et al., 2013; Morgan et al., 2014). Such tests need to be valid, reliable, cost-effective, and easy to implement in a sports setting (Garrick, 2004; Maffey and Emery, 2006). The sit and reach test (S&R), adductor squeeze test (AST), and dorsiflexion lunge test (DLT) are examples of commonly used tests in repeated-measures screening designed to provide measures of lower back and hamstring flexibility, hip adductors' strength, and calf flexibility (through ankle dorsiflexion range of motion) respectively (Bennell et al., 1998; Gabbe et al., 2004; Malliaras et al., 2009). These tests have good to excellent intra-tester reliability with intraclass correlation coefficients (ICC) between 0.81 and 0.98 (Bennell et al., 1998; Gabbe et al., 2004; Malliaras et al., 2009). The standard error of measurement (SEM) was calculated as 1 cm for the S&R, 0.5 to 0.6 cm for the DLT, and 20 mmHg (~10%) for the AST (Bennell et al., 1998; Gabbe et al., 2004; Malliaras et al., 2009). However, these reliability measures have been calculated for only two measurements with test-retest gaps between 30 min and 1 week, and it is not clear to what extent regular exposure to training and competition over extended periods affects these measures. Understanding the normal variability of test scores throughout the season, when athletes are not injured, is a crucial step in identifying the relationship between the changes in test scores, maladaptation to training, and the risk of injuries (Bakken et al., 2016).

Accumulation of training-induced stress on the musculoskeletal system may result in maladaptation and increased risk of injuries (Vanrenterghem et al., 2017). In the absence of direct measurement methods of biomechanical load on body tissues in a field context, indirect methods such as the session rating of perceived exertion (sRPE) have been proposed as viable alternatives (Vanrenterghem et al., 2017). Musculoskeletal measures respond to the acute load of soccer and Australian football matches (Dawson et al., 2005; Paul et al., 2014); thus, it is also important to investigate the effects of training load on the possible changes in the test scores. In addition, individual differences in the normal variability requires investigation in order to develop an effective flagging system based on the changes in scores relative to their normal variability. The aim of this study was to identify the normal variability of a selection of weekly musculoskeletal screening tests and the associated individual differences in variability, as well as the influence of training load on the changes in test scores across an Australian football season.

## Methods

### Participants

All the 44 elite male players from one Australian football club were invited and agreed to participate in this study (mean age ± *SD*; 22.8 ± 4.0). The study was approved by Victoria University Human Research Ethics Committee, and all participants provided written informed consent in accordance with the Declaration of Helsinki.

### Study design

Weekly musculoskeletal screening scores and daily internal training load were recorded for individual players over an entire AFL season. Weekly musculoskeletal screening tests were conducted within 3 h prior to the first field training session of the week which was planned 2 or 3 days apart from a previous field training session or a match. Based on the club's training schedule, screening occurred on Monday mornings during pre-season and Tuesday afternoons during in-season. This timing was chosen to allow the medical staff to further investigate players with abnormally reduced scores or accompanying symptoms prior to the training session. Pre-season and in-season periods were analyzed separately due to the possible effects of diurnal variation (Manire et al., 2010). The final 5 weeks of the official pre-season involved match simulations and a pre-season tournament during which the training schedule, training loads, and screening times resembled those of the in-season. As a result, this phase was considered as a part of the in-season for the purposes of this study. Thirty-five screening sessions were held in total (pre-season = 8, in-season = 27) with no screening on some other weeks due to team unavailability (Christmas break, training camp, and scheduling issues). Individual screening scores were excluded from the analysis when a player was diagnosed as injured by the club's medical staff and could not fully participate in the training session that followed the screening.

### Screening tests

#### Sit and reach test

Players placed their bare feet against the sit and reach box and their middle fingers on top of each other. They were then asked to stretch forward as far as possible and hold the position for 1 s while keeping the knees straight. The reach distance from the tip of the middle fingers relative to the toe line was recorded (Gabbe et al., 2004).

#### Dorsiflexion lunge test

A permanent tape measure was fixed on the floor with 0 cm mark at a wall junction. Players were asked to place the big toe and heel of the testing leg beside the tape. They were then instructed to lunge forward until the knee touches the wall while keeping the heel in contact with the floor. The maximum distance from the tip of the big toe to the wall was recorded (Bennell et al., 1998).

#### Adductor squeeze test

With players in a supine position, a sphygmomanometer cuff pre-inflated to 20 mmHg was placed between the knees. Players were asked to maximally squeeze the cuff and hold for 1 s and the maximum pressure displayed on the dial was recorded. The test was conducted in three hip flexion angles of 0°, 45°, and 90° (Malliaras et al., 2009).

### Training load

The session rating of perceived exertion (sRPE) method was used to quantify the individual internal training load for all training sessions and matches (RPE multiplied by the session duration) (Foster, 1998). The sRPE method has been validated for monitoring training load in Australian football (Scott et al., 2013). Various cumulative and relative measures of training load were then calculated with each screening day as the reference point. These measures included the 7, 14, 21, and 28 day cumulative loads; monotony; strain; acute to chronic load ratio (mean daily load of the past 7 days divided by the mean daily load of the past 28 days); and the smoothed load (Foster, 1998; Hulin et al., 2016). The smoothed load is an exponentially weighted moving average of training load, which accounts for the decaying effects of training load using a decay factor λ (lambda) (Hunter, 1986; Williams et al., 2017). The smoothed load at the beginning of each day is calculated as [λ × (yesterday's training load)] + [(1 – λ) × the smoothed load up to that point]. The decay factor λ defines a time constant 1/λ representing the period that contains ~2/3 of the total weighting in calculation of the smoothed load. The smoothed load was calculated with decay factors of 0.33, 0.14, 0.07, and 0.036 representing time constants of 3, 7, 14, and 28 days respectively. It should be noted that our method of labeling the time constants (1/λ) is slightly different to the one recently suggested [(2- λ)/λ] (Williams et al., 2017). Using our method of labeling the time constant, the smoothed load of a given period has the highest correlation with the simple cumulative load of a similar period (Table 1).

**Table 1:** Correlations between cumulative and smoothed training loads of various periods on a given day^a^.

	**Cumulative 3 day**	**Cumulative 7 day**	**Cumulative 14 day**	**Cumulative 21 day**	**Cumulative 28 day**
**PRE-SEASON**^b^					
Smoothed 3 day	**0.91**	0.81	0.63	0.39	0.18
Smoothed 5 day	0.83	**0.91**	0.81	0.58	0.33
Smoothed 7 day	0.73	**0.90**	0.89	0.71	0.46
Smoothed 10 day	0.60	0.81	**0.91**	0.82	0.61
Smoothed 14 day	0.46	0.67	**0.86**	**0.86**	0.72
Smoothed 21 day	0.32	0.48	0.68	**0.77**	0.75
Smoothed 28 day	0.24	0.36	0.52	0.62	**0.65**
**IN-SEASON**^c^					
Smoothed 3 day	**0.85**	0.70	0.59	0.48	0.42
Smoothed 5 day	0.81	**0.82**	0.77	0.66	0.59
Smoothed 7 day	0.75	0.84	**0.86**	0.78	0.72
Smoothed 10 day	0.66	0.81	**0.90**	0.87	0.83
Smoothed 14 day	0.57	0.75	0.90	**0.92**	0.90
Smoothed 21 day	0.46	0.66	0.84	0.90	**0.93**
Smoothed 28 day	0.40	0.58	0.77	0.86	**0.91**

a *Values are Pearson correlation coefficients. The highest value of each row is in bold*.

b *The number of observations for training load measures ranged from 3,271 (cumulative 28 day) to 4,503 (smoothed loads)*.

c *The number of observations was 8800 for each training load measure. Unlike the pre-season phase, all measures could be calculated from the first day of the in-season*.

### Statistical analysis

The analyses were performed in three parts using the Statistical Analysis System (version 9.4, SAS Institute, Cary, NC). Based on the scale of the test scores, only the AST scores were log-transformed before modeling (Hopkins et al., 2009). In the first part, each individual's within-subject variability of test scores in each season phase was derived separately as the standard error of the estimate (SEE) of the scores using a general linear mixed model that included a linear trend over each phase. The mean of the individual SEEs represented the normal variability of the scores over each phase. The individual SEEs were then analyzed in a meta-analytic mixed model with a random effect representing true differences between the individual SEEs and expressed as a factor *SD*. The difference between individuals with typically high variability (mean SEE × factor *SD*) and low variability (mean SEE ÷ factor *SD*) was used to assess the magnitude of the individual differences in variability (Smith and Hopkins, 2011; Hopkins, 2015).

In the second part, another general linear mixed model was devised to identify any possible linear trends in the scores at each phase by including the week as a numeric fixed effect. The week number and player identity were defined as nominal random effects. A model in which a different variability (the residual) was specified for each player failed to converge for any of the tests. To account for the real differences in variability, the players were therefore assigned to three subgroups of low, moderate, and high variability based on the findings of the previous part, with a separate residual for each subgroup. A dummy variable for the number of days post-match that the screening occurred (two or three) was added to the model. This dummy variable was used to compare the within-subject differences in the scores as a result of an extra recovery day post-match.

In the third part, a quadratic mixed model was developed to evaluate the effects of various measures of training load on the screening scores. The intercept, training load measure, and the square of the training load measure were the fixed effects which collectively estimated the mean quadratic. The random effects were player identity (to estimate different between-player means across each season phase), the interaction of player identity with the training measure and with the square of the training measure (to estimate individual differences in the players' quadratics), and the residual error (within-player week to week variability). This model estimated the within-subject changes in a given screening score associated with within-subject changes in a given measure of training load. Within-player *SD*s of training load in each season phase were therefore used to estimate the magnitude of effects. The scores were estimated at typically very low (−2*SD*), low (−1*SD*), mean, high (+1*SD*), and very high (+2*SD*) values of training load. On the few occasions where −2*SD* of training load was a negative value, the estimates for the screening scores were calculated for zero training load. Uncertainty in the estimate of the turning point of the quadratic curve was determined via parametric bootstrapping (Hébert-Losier et al., 2015). The turning points were mostly unclear (>10% of the bootstrap samples had quadratic curvature opposite to the observed curvature) because the effect of training on the test scores was approximately linear. Hence, a 2*SD* difference in the predictor (from −1*SD* to +1*SD*) was used to quantify the magnitude of the effects of training load (Hopkins et al., 2009).

The findings were interpreted using mechanistic magnitude-based inference (Hopkins et al., 2009). The uncertainty in estimates was expressed as 90% confidence limits (CL) and qualitatively as chances that the true value of the estimate was either trivial or substantial (larger than the smallest important change) using the following scale: <0.5%, most unlikely; 0.5% to <5%, very unlikely; 5% to <25%, unlikely, 25% to <75%, possibly; 75% to <95%, likely; 95% to <99.5%, very likely; >99.5%, most likely. The true change was deemed unclear when the chances of substantial positive and negative change were both >5% (Hopkins et al., 2009). The smallest important change for the AST was calculated as 0.2 of the observed between-subject *SD* (Hopkins et al., 2009). The raw S&R and DLT scores are influenced by anthropometry, and differences between individuals may not be due to real differences in flexibility and range of motion (Hopkins and Hoeger, 1992; Bennell et al., 1998). Consequently, a smallest important change of 1 cm was selected for these tests, based on clinical experience. Smallest important changes were halved for interpretation of magnitude of *SD*s representing variability (Smith and Hopkins, 2011; Hopkins, 2015). Changes representing trivial, small, moderate and large magnitudes were consistent with those provided by standardization (<1x, 1x, 3x, and 6x the smallest important change respectively) (Hopkins et al., 2009).

## Results

The findings for the left and right DLTs were nearly identical as were the findings for the three ASTs. Hence, only the results for the right DLT and AST at 0 degrees of hip flexion are shown. One player sustained a season-ending injury at the end of pre-season and was excluded from the in-season analysis. Table 2 summarizes the statistics derived from the first and second parts of the analysis. Substantial small to moderate variability was found for all the tests at both pre-season and in-season when players were cleared to fully participate in the training session that followed the screening. Likely to very likely small individual differences in variability existed for the S&R and AST. The only substantial trend was a very likely small increase in the AST over the in-season. Not shown in the table are the differences between the scores when the screening was conducted at 3 vs. 2 days post-match (Saturday vs. Sunday match); these were all most likely trivial (for example, the difference for the AST was −0.6%, 90% CL ±1.2%).

**Table 2:** Statistics summarizing screening test scores for an AFL team in a pre- and in-season phase derived from parts 1 and 2 of the analysis.

	**Statistics from reliability analysis allowing for linear trend in each phase**			**Statistics from meta-analysis of individual values of within-subject variability (SEE)**	
	**Mean ± between-subject *SD* in a typical test**	**Trend over the season phase; ±90%CL**	**Intraclass correlation; ±90%CL**	**Mean variability; ±90%CL**	**Individual differences in variability as factor SD^a^; × /÷90%CL**
**SIT AND REACH TEST (cm)**					
Pre-season	2.3 ± 8.2	0.21; ± 0.24^ML↔^	0.98; ± 0.01	0.92; ± 0.14^ML↕^	1.66; × /÷1.12 ^VL↕^
In-season	3.1 ± 7.9	0.03 ± 0.23^ML↔^	0.97; ± 0.01	1.01; ± 0.12^ML↕^	1.52; × /÷1.09 ^VL↕^
**DORSIFLEXION LUNGE TEST (cm)**					
Pre-season	11.4 ± 3.2	−0.05; ± 0.14^ML↔^	0.97; ± 0.01	0.50; ± 0.08^P↕^	1.43; × /÷1.15 ^L↔^
In-season	11.3 ± 3.3	−0.05; ± 0.09^ML↔^	0.96; ± 0.01	0.48; ± 0.06^P↕^	1.37; × /÷1.10 ^L↔^
**ADDUCTOR SQUEEZE TEST**					
Pre-season	253 mm Hg ± 20%	0.4%; ± 2.9%^ML↔^	0.74; ± 0.08	7.8%; ± 0.8%^ML↕↕^	1.38; × /÷1.10 ^VL↕^
In-season	266 mm Hg ± 21%	6.5%; ± 2.2%^VL↑^	0.81; ± 0.07	7.4%; ± 0.6%^ML↕↕^	1.31; × /÷1.07 ^L↕^

a *Multiply and divide the mean variability by this factor to get typically high and low individual values of the variability*.

*AFL, Australian football league; SD, standard deviation; SEE, standard error of the estimate; CL, confidence limits*.

*Likelihood: P, possibly; L, likely; VL, very likely; ML, most likely*.

*Magnitude of variability: ↔ trivial; ↕ small; ↕↕ moderate*.

*Magnitude of trend: ↔ trivial; ↑ small increase*.

**Figure 1:**
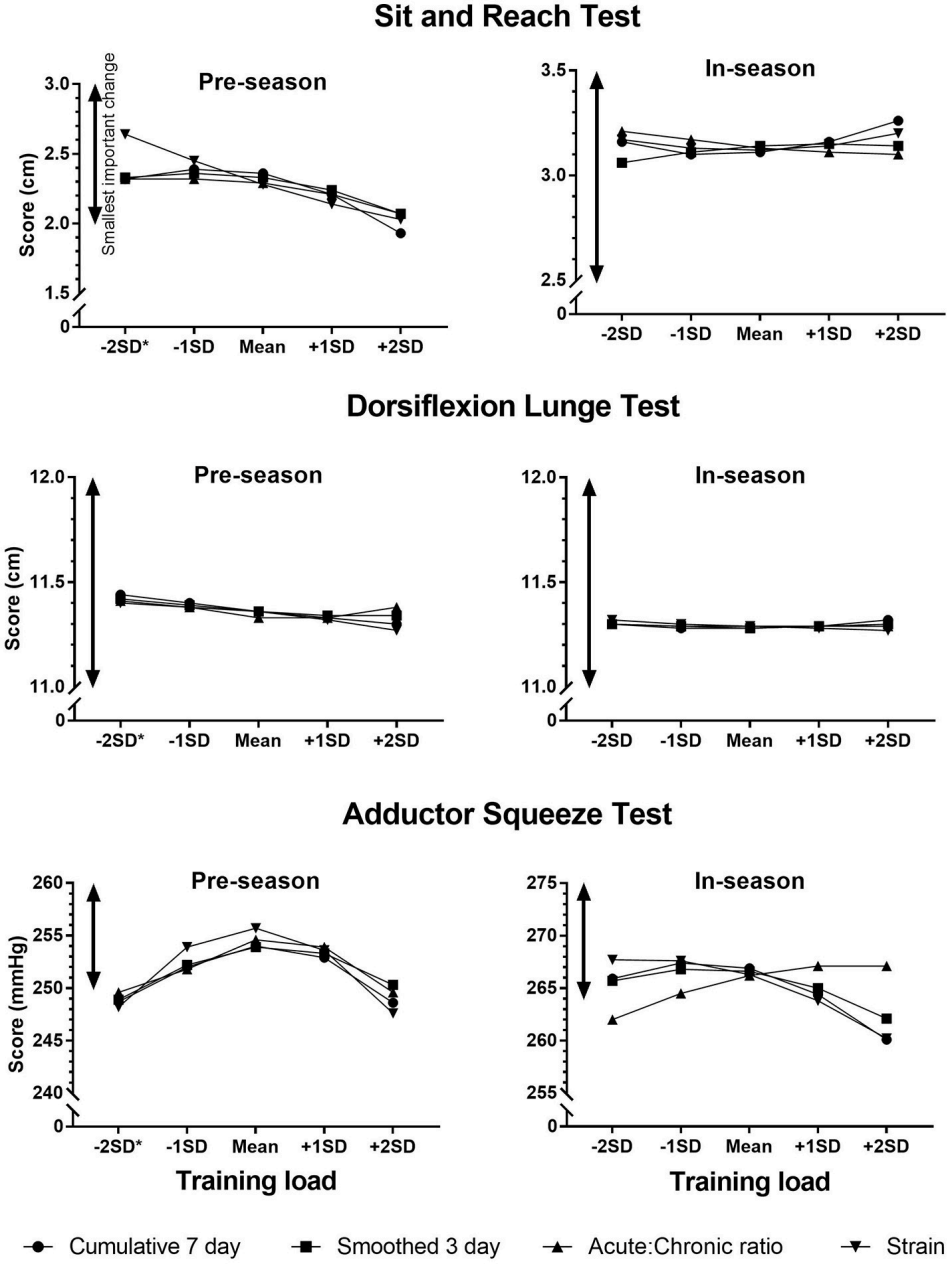
Changes in screening scores with changes in training load (cumulative 7 day, smoothed 3 day, acute:chronic ratio, strain). ^*^The estimates for the screening scores were calculated for zero training load where −2*SD* of training load was a negative value.

**Figure 2:**
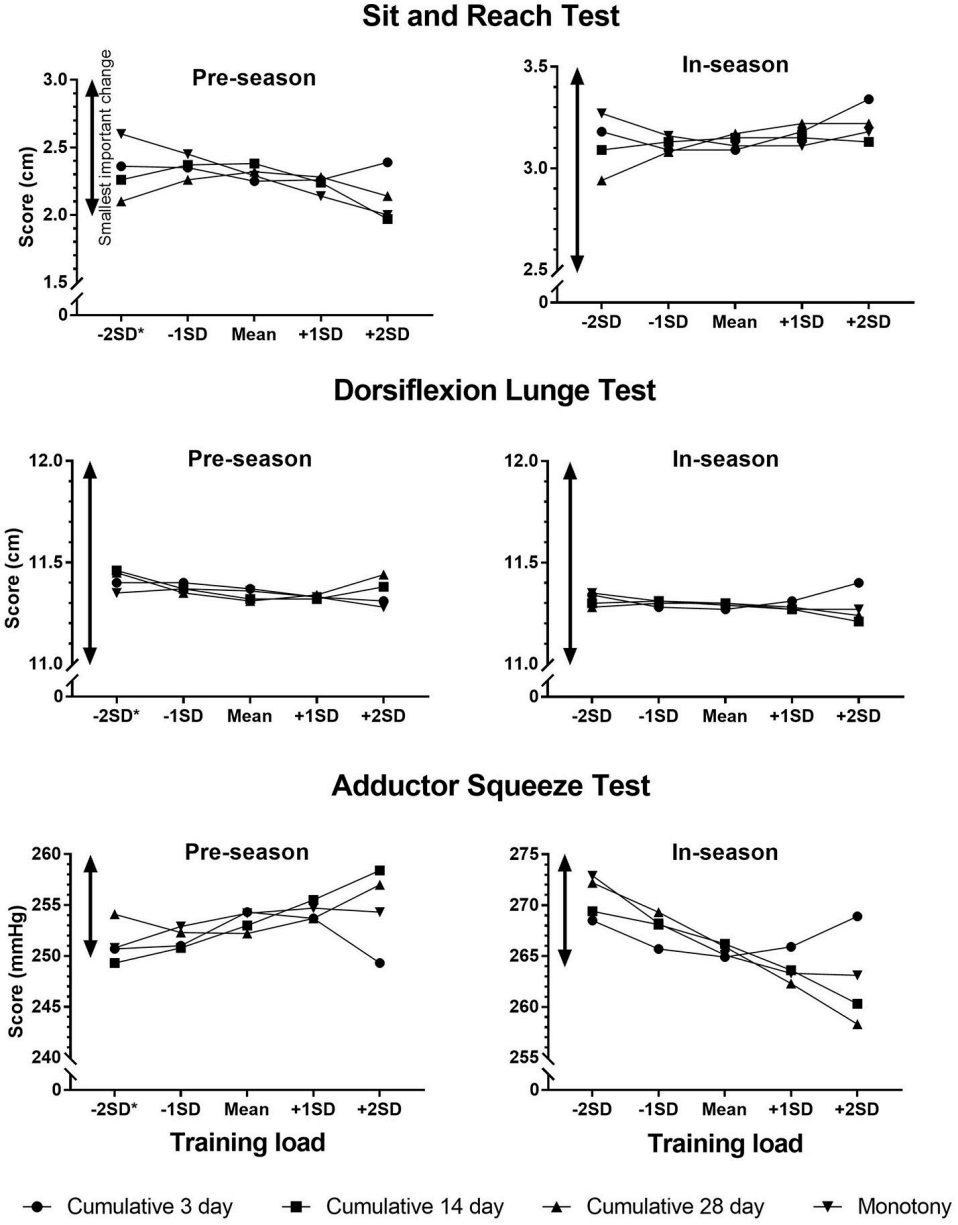
Changes in screening scores with changes in training load (cumulative 3 day, cumulative 14 day, cumulative 28 day, monotony). ^*^The estimates for the screening scores were calculated for zero training load where −2*SD* of training load was a negative value.

**Figure 3:**
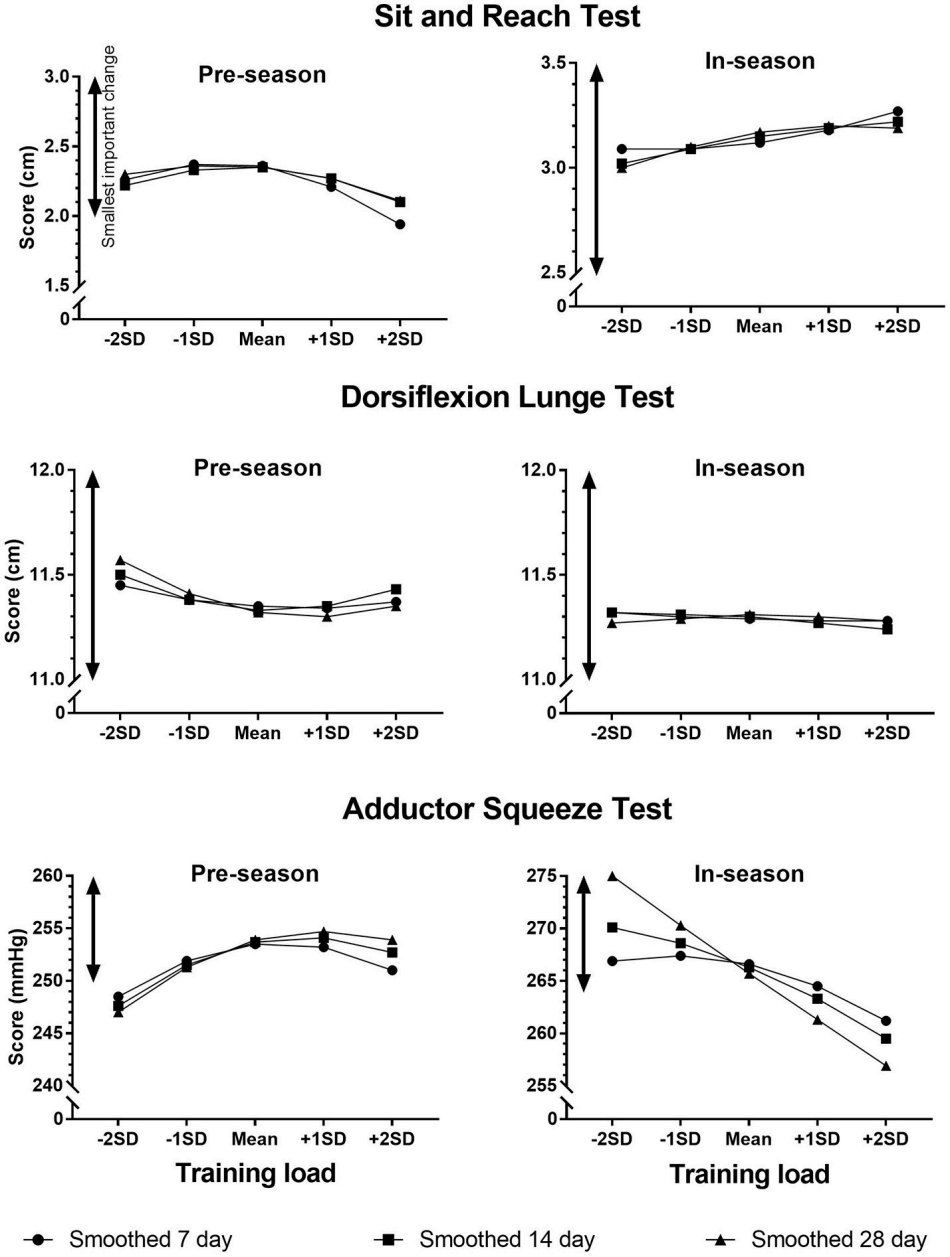
Changes in screening scores with changes in training load (smoothed 7 day, smoothed 14 day, smoothed 28 day).

The effects of an increase in training load from −1*SD* to +1*SD* on the screening scores are shown in Table 3. Figures 1 – 3 show the changes in screening scores with changes in training load over a wider range (−2*SD* to +2*SD*). All measures of training load had trivial effects on the screening scores at both pre-season and in-season.

**Table 3:** Effects of training load on the test scores derived from part 3 of the analysis.

**Training load measure**	**Mean ± within–subject *SD***	**Effect of an increase in training load from** −**1*****SD*** **to** +**1*****SD***^a^		
		**S&R; ± 90% CL (cm)**	**DLT; ± 90% CL (cm)**	**AST; ± 90% CL (%)**
**PRE–SEASON (*****n*** = **44)**				
Cumulative 3 day	410 ± 380	−0.09; ± 0.17^ML↔^	−0.07; ± 0.07^ML↔^	1.1; ± 1.6^ML↔^
Cumulative 7 day	2, 440 ± 1, 260	−0.18; ± 0.20^ML↔^	−0.07; ± 0.10^ML↔^	0.4; ± 1.9^ML↔^
Cumulative 14 day	4, 260 ± 2, 160	−0.13; ± 0.20^ML↔^	−0.05; ± 0.10^ML↔^	1.8; ± 1.9^VL↔^
Cumulative 28 day	8, 120 ± 2, 300	0.02; ± 0.17^ML↔^	−0.01; ± 0.05^ML↔^	0.6; ± 1.8^ML↔^
Smoothed 3 day	210 ± 120	−0.12; ± 0.19^ML↔^	−0.05; ± 0.09^ML↔^	0.3; ± 1.9^ML↔^
Smoothed 7 day	280 ± 120	−0.16; ± 0.22^ML↔^	−0.04; ± 0.10^ML↔^	0.5; ± 2.0^VL↔^
Smoothed 14 day	300 ± 90	−0.06; ± 0.18^ML↔^	−0.03; ± 0.10^ML↔^	1.0; ± 1.8^VL↔^
Smoothed 28 day	330 ± 90	−0.09; ± 0.20^ML↔^	−0.11; ± 0.15^ML↔^	1.4; ± 2.0^VL↔^
Acute:Chronic ratio	1.20 ± 0.80	−0.11; ± 0.17^ML↔^	−0.05; ± 0.07^ML↔^	0.8; ± 1.7^ML↔^
Monotony	0.86 ± 0.22	−0.30; ± 0.25^ML↔^	−0.04; ± 0.10^ML↔^	0.7; ± 2.2^VL↔^
Strain	2, 560 ± 1, 150	−0.30; ± 0.21^ML↔^	−0.07; ± 0.09^ML↔^	−0.1; ± 1.7^ML↔^
**IN–SEASON (n = 43)**				
Cumulative 3 day	950 ± 360	0.08; ± 0.14^ML↔^	0.03; ± 0.05^ML↔^	0.1; ± 1.1^ML↔^
Cumulative 7 day	1, 750 ± 340	0.05; ± 0.13^ML↔^	0.01; ± 0.04^ML↔^	−1.1; ± 0.9^ML↔^
Cumulative 14 day	3, 510 ± 530	0.02; ± 0.11^ML↔^	−0.05; ± 0.05^ML↔^	−1.7; ± 0.9^ML↔^
Cumulative 28 day	7, 080 ± 980	0.14; ± 0.15^ML↔^	−0.02; ± 0.08^ML↔^	−2.6; ± 1.3^L↔^
Smoothed 3 day	250 ± 60	0.04; ± 0.15^ML↔^	0.00; ± 0.05^ML↔^	−0.7; ± 1.0^ML↔^
Smoothed 7 day	260 ± 40	0.09; ± 0.12^ML↔^	−0.02; ± 0.05^ML↔^	−1.1; ± 0.9^ML↔^
Smoothed 14 day	260 ± 30	0.10; ± 0.13^ML↔^	−0.04; ± 0.06^ML↔^	−2.0; ± 0.9^ML↔^
Smoothed 28 day	260 ± 30	0.09; ± 0.19^ML↔^	0.01; ± 0.13^ML↔^	−3.3; ± 1.6^P↔^
Acute:Chronic ratio	1.0 ± 0.19	−0.06; ± 0.15^ML↔^	0.00; ± 0.04^ML↔^	1.0; ± 0.9^ML↔^
Monotony	0.82 ± 0.20	−0.04; ± 0.15^ML↔^	−0.04; ± 0.08^ML↔^	−1.8; ± 1.2^ML↔^
Strain	1, 430 ± 430	0.01; ± 0.13^ML↔^	−0.02; ± 0.07^ML↔^	−1.4; ± 1.2^ML↔^

a *All effects were trivial. S&R, sit and reach test; DLT, dorsiflexion lunge test; AST, adductor squeeze test*.

*SD, standard deviation; CL, confidence limits*.

*↔ trivial; P, possibly; L, likely; VL, very likely; ML, most likely. Cumulative, smoothed, and strain values are in arbitrary units*.

## Discussion

There were substantial small to moderate amounts of normal variability with some individual differences in variability associated with the weekly musculoskeletal screening tests. The tests which were conducted two or three days post-match (or main training session during pre-season) were not sensitive to changes in internal training load and may not provide an accurate indication of the athletes' readiness for training when used as measures of recovery.

### Normal variability

This study is the first to have tracked weekly test scores throughout an entire season. The intra-tester reliability of the tests in the current study as quantified using ICC, were similar to those in studies with test-retest gaps of between 30 min and 1 week (Bennell et al., 1998; Gabbe et al., 2004; Malliaras et al., 2009). The normal variability of the test scores was approximately ± 1.0 cm for the S&R, ± 0.5 cm for the DLT, and ± 8% for the AST. These values are similar to the previously reported SEMs (Bennell et al., 1998; Gabbe et al., 2004; Malliaras et al., 2009) and do not seem to be affected by regular exposure to training and competition throughout the season. Such stability in reliability despite physical challenges of a long competitive season indicate that substantial changes in weekly scores cannot be simply attributed to training-induced altered reliability of the tests. Various sources such as technique variation, equipment error, and true change in athletes' test performance contribute to the week to week changes in screening scores (Hopkins, 2000). The true change in test performance itself may arise from adaptation or maladaptation to training and competition, the residual effects or complete resolution of a previous injury, or minor incidents that affect the test scores without limiting the athletes' capacity to fully participate in training (e.g., minor muscle contusions). Thus, it is important for clinicians to interpret the findings of weekly screening in light of possible contributing factors toward the change in the scores.

The typical error (noise) obscures the important change (signal) in any measure (Hopkins, 2000). In the concept of weekly screening, noise is represented by the normal variability of the scores as measured in the current study. The signal can be considered as the smallest change in the screening score that is associated with a substantial increase in the risk of injury. Reductions of ~12% and 6% in the hip adductors' strength of elite junior Australian footballers (as measured by a hand-held dynamometer) were reported during the week of groin injury onset and the preceding week respectively, which represent the signal for that particular test (Crow et al., 2010). No studies to date have evaluated the signal for either of the tests for which we established the noise. Future studies investigating the signal should take into account the normal variability of the test scores throughout the season when interpreting the findings and assessing the potential of weekly screening tests for injury prevention purposes.

### Individual differences in variability

Training, like any intervention, interacts with the athletes' individual characteristics making the effects more or less beneficial, harmful, or ineffective in different individuals (Hopkins, 2015). In the case of weekly screening, such interactions led to the observed individual differences in variability which were substantial for the S&R and AST (Table 2). For instance, the S&R score in players with typically low normal variability (1*SD* below the mean) varied by approximately ± 0.5 cm from one week to another week, while players with typically high normal variability (1*SD* above the mean) showed a typical week to week variation of approximately ± 1.5 cm. Applying an arbitrary threshold to the change in screening scores for flagging purposes may prove overly sensitive for some players and not sensitive enough for others.

A survey of athlete monitoring practices in high performance sports revealed that the majority of coaching and support staff rely on visual identification of trends in the athletes' data to identify the ones who may benefit from an adjustment to training load (Taylor et al., 2012). Another common method was the use of red flags with thresholds being set by either arbitrary cut-off points or within-subject *SD*s (Taylor et al., 2012). On the basis of the observed individual differences in the current study and previous recommendations on the development of decision support systems (Robertson et al., 2017), we encourage the use of within-subject *SD*s in setting the flagging thresholds for weekly musculoskeletal screening. In the absence of enough longitudinal data when within-subject *SD*s cannot be reliably estimated, practitioners may use the reported normal variations to detect abnormal changes in the screening scores.

### Effects of training load

The observed trivial effects of training load on the test scores indicate that these tests are not sensitive to changes in internal training load when performed 2 or 3 days post-match or post-training. Subsequently, the screening scores should be interpreted cautiously when used as measures of recovery. This finding is supported by the observed trivial differences between the test scores obtained at 2 vs. 3 days post-match in the current study as well as the previously reported timeline of change in the measures of flexibility and peak force post-match (Dawson et al., 2005; McLellan et al., 2011; Johnston et al., 2013; Roe et al., 2016; Wollin et al., 2017). The S&R score declines on day 1 post-match and returns to baseline on day 2 in elite Australian footballers (Dawson et al., 2005). Measures of lower limb strength return back to baseline by day 1 post-match (McLellan et al., 2011; Wollin et al., 2017) or do not change in the first place in team sport athletes (Johnston et al., 2013; Roe et al., 2016).

Training load has an established association with the recovery of athletes and injury risk (Gabbett, 2016). In the absence of a substantial relationship between training load and screening scores, a normal test score does not necessarily mean that the athlete is sufficiently recovered to process another training stimulus, and other more sensitive measures of recovery should be evaluated by practitioners. On the other hand, an abnormal screening score is often indicative of an underlying issue that needs to be investigated by the medical staff prior to the training session. There are also other benefits associated with musculoskeletal screening which include identifying undiagnosed injuries or complaints, assessing the rehabilitation progression of previous injuries, establishing future return-to-play outcome measures for healthy athletes, and establishing rapport between the medical staff and athletes (Bahr, 2016; Bakken et al., 2016; Clarsen and Moseby Berge, 2016).

Overall, while weekly musculoskeletal screening appears to be a valuable athlete monitoring tool, clinicians need to be aware of the normal variability of the test scores and the individual differences in such variability when interpreting changes in screening scores. The lack of sensitivity of the investigated tests to training load should prompt clinicians to investigate the reasons behind substantial reductions in screening scores rather than casually attributing them to a match or training session that occurred more than 2 days prior to the screening.

A limitation of this study is that the current findings in regards to the effects of training load on the musculoskeletal screening scores are based on the sRPE derived internal measures of training load and may not necessarily apply to the external measures of training load (e.g., running distance). Considering the differences between adaptation pathways to physiological and biomechanical loads (Vanrenterghem et al., 2017), future studies need to investigate the relationship between external measures of training load and the response of the musculoskeletal system. To the best of our knowledge, the screening results on a given day did not generally change the injury status of players on the day of screening. However, as a general limitation of working with elite athletes, the implemented interventions in response to the screening scores (e.g., additional treatment sessions) could have affected the screening scores in the following week. It should also be noted that the current study was conducted with elite male Australian footballers, and generalization of findings to females as well as athletes of other sports and levels of play should be done with caution.

## Conclusion

A change in the screening scores larger than the identified normal variability is required to be considered a true change and the flagging systems applied to the screening scores need to account for the individual differences in variability. The studied tests are not sensitive to changes in training load as the scores return back to baseline by day 2 post-match or post-training when the screening is normally conducted.

## Author contributions

AE, RA, AS, WH, GE: conceived and designed the study; AE: performed the tests; WH, AE, BL, AR: analyzed the data; AE, WH, RA, AS: interpreted the results; AE: drafted the manuscript and prepared the tables/figures; AE, RA, WH, BL, AR, GE, AS: edited, critically revised the manuscript, and approved the final version.

### Conflict of interest statement

The authors declare that the research was conducted in the absence of any commercial or financial relationships that could be construed as a potential conflict of interest.
